# Epidemiology of Congenital Heart Disease in Jinan, China From 2005 to 2020: A Time Trend Analysis

**DOI:** 10.3389/fcvm.2022.815137

**Published:** 2022-04-27

**Authors:** Lihua Zhang, Bei Liu, Huimin Li, Chengxiang Wang, Shimin Yang, Zhongliang Li

**Affiliations:** ^1^Department of Medicine, Jinan Maternity and Child Care Hospital Affiliated to Shandong First Medical University, Jinan, China; ^2^Department of Health Education, Jinan Health Publicity and Education Center, Jinan, China; ^3^Neonatal Intensive Care Unit, Jinan Maternity and Child Care Hospital Affiliated to Shandong First Medical University, Jinan, China; ^4^Department of Women Healthcare, Jinan Maternity and Child Care Hospital Affiliated to Shandong First Medical University, Jinan, China; ^5^Department of Public Health, Jinan Maternity and Child Care Hospital Affiliated to Shandong First Medical University, Jinan, China

**Keywords:** epidemiology, congenital heart disease, prevalence, time trend analysis, causes analysis

## Abstract

**Background:**

Although congenital heart defect (CHD) was the dominating birth defect, the time trend analysis of CHD was largely unknown. In our study, the time trend analysis of CHD from 2005 to 2020 in Jinan was conducted, aimed to reveal the epidemiological characteristics in a city and provided the data basis for the government to make a policy intervention.

**Methods:**

A multi-institutional and retrospective review of CHD for all births from January 1, 2005 to December 31, 2020 was performed. Proportioner prevalence was used to describe the distribution of CHD. Comparisons of CHD characteristics among different groups were assessed with Chi-squares tests. Cochran-Armitage tests (CAT) were used to track changes in CHD prevalence.

**Results:**

About 322,374 births and 5,180 CHD in Jinan were included from 2005 to 2020, and the total CHD prevalence was 3.92 per 1,000 births. The CHD prevalence showed an upward trend, with a total increase of 227.66% from 2005 to 2020. The CHD prevalence in urban areas was 34.17% higher than that in rural areas, but the gap was narrowing. Atrial septal defect (3.07 per 1,000 births), patent ductus arteriosus (1.62 per 1,000 births), ventricular septal defect (1.18 per 1,000 births), tetralogy of Fallot (0.62 per 1,000 births), and atrioventricular septal defect (0.47 per 1,000 births) were the 5 most common subtypes.

**Conclusion:**

The prevalence of CHD in Jinan was gradually on the rise, which needs to be highly focused on by the health management department. Older pregnant women and women in rural areas should be concerned, and targeted measures need to be introduced.

## Introduction

Congenital heart disease (CHD) was defined as clinically significant structural heart and/or great vessels disease present at birth (1). It was estimated that the global average prevalence of CHD was 9.4 per 1,000 live births in 2010–2017 (2). CHD accounts for 28% of all birth defects, which was the most common birth defect (3). In China, 90–150 thousand new births with CHD were diagnosed every year, and infant mortality due to CHD reached 3.91 per 1,000 births in 2018 (4). Although child mortality caused by CHD has declined, the burden of disease caused by CHD was still serious in developing countries (5). CHD was also an important cause of disability in children (6). Infants with CHD frequently required early surgical treatment after birth to improve outcomes (7, 8), which always caused serious economic burden. The annual healthcare costs associated with CHD were estimated to be $5.6 billion, accounting for 15.1% of total pediatric hospitalization costs (9). In China, the total economic burden of CHD exceeded 12.6 billion RMB every year (10). In view of the heavy disease burden and serious health damage caused by CHD, the comprehensive prevention and control of CHD had become a public health issue. Therefore, the reliable epidemiological characteristics of CHD were important in order to have a better insight into its etiology and a finding of high-risk population.

Many studies in China have reported the prevalence of CHD. However, the period for identifying CHD in most studies was from 28 gestation weeks to 7 days after births. In fact, as with the large-scale use of ultrasound in obstetrics, more and more CHDs were diagnosed before 28 gestation weeks. So, that monitoring period cannot reflect the real level of CHD.

Jinan is the most representative city in eastern China for its geographic character, economic figure, and composition of population. In 2005, Jinan established a birth defect monitoring network, covering all medical institutions in the city. In 2011, all maternal and newborn information, including information on CHD, was reported through the web-based reporting system, and accurate and reliable data were available.

In this study, we extended the monitoring period of CHD, from 12 gestation weeks to 7 days after birth. We also performed a trend analysis and a difference test on the characteristics and subtypes of CHD, and the causes of these results were also analyzed. We believe that our study would provide suggestions for the government to formulate scientific policies and serve as a model for some developing cities to prevent CHD.

## Methods

### Study Population

A multi-institutional and retrospective review of CHD for all births from January 1, 2005 to December 31, 2020 was performed. The study population involved all the pregnant women and fetus/neonates in hospitals of Jinan. The period of identifying CHD was from 12 gestation weeks to 7 days after births, during which CHD diagnosed for the first time was required to report. CHD was coded according to the International Classification of Diseases (10th edition) (11).

### Data Collection

Data collection was based on the three-level network of the Maternal and Child Healthcare Institutions (MCH), including hospitals, county-level MCH, and city-level MCH (12). The doctors in hospitals reported the information of pregnant women and fetus/neonates to county-level MCH. The submitted data were verified by the staff in county-level MCH and reported to the city-level MCH every month. Ultimately, 52 hospitals, including 1,322,374 births and 5,180 CHD, were included in the study.

Variables such as the diagnosis gestation week of CHD, the gender of CHD, the maternal age of CHD, the subtype of CHD, and the other demographic characteristics were collected and analyzed.

### Data Quality Management

In order to keep high-quality data, a series of standard operating procedures, including data collection, data abstraction, data evaluation, were developed. To ensure accuracy and completeness of CHD, quality control was routinely performed, and 10% normal findings and all abnormal findings were reviewed by cardiologists.

In the process of quality control, the related variables of CHD, such as the gestation week, the CHD subtype, the maternal age, and so on, were the key check contents.

### Statistical Analysis

Demographic and clinical characteristics of CHD were expressed as proportion or prevalence. Cochran-Armitage tests (CATs) were used to track changes of the prevalence of CHD in rural and urban areas, the proportion of CHD before and after 28 gestation weeks, and the prevalence of CHD subtypes. The prevalence of CHD according to different maternal characteristics was assessed with Chi-squares tests. Cochran-Armitage tests (CATs) were used to track changes of the CHD prevalence. R version 3.5.1 (the Comprehensive R Archive Network) was used for the data analysis, with a significance level at *p* < 0.05.

## Results

### The Prevalence of CHD in Jinan From 2005 to 2020

We counted 1,322,374 births and 5,180 CHD from 2005 to 2020, and the prevalence was 3.92 per 1,000 births. Time trend analysis showed that the prevalence of total CHD was increased, from 1.52 per 1,000 births in 2005 to 4.99 per 1,000 births in 2020, with a total increase of 227.66% and an annual increase of 14.23%.

**Figure 1:**
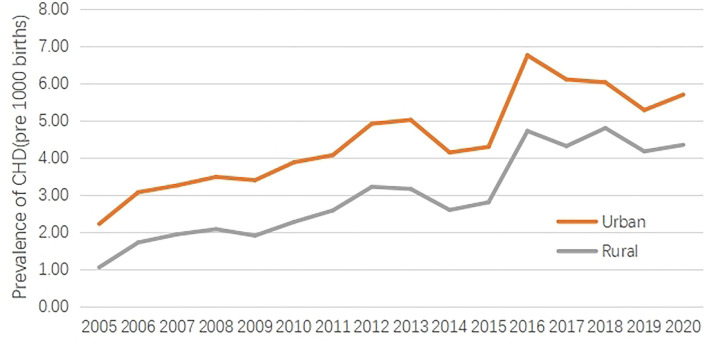
The prevalence of CHD between urban areas and rural areas from 2005 to 2020.

Overall, during the study period, an “S” shape curve can be found in the time trend of CHD prevalence. The first stage was from 2005 to 2010, during which the CHD prevalence was about 2.46 per 1,000 births. The second stage was from 2011 to 2015, during which the CHD prevalence was about 3.58 per 1,000 births. The third stage was from 2016 to 2020, during which the CHD prevalence was about 5.21 per 1,000 births (Table 1, Figure 1).

**Table 1:** The prevalence of CHD between urban areas and rural areas from 2005 to 2020.

**Year**	Urban^*^			**Rural^&^**			**Total^#^**			**Extent%Δ^$^**
	**Births**	**CHD**	**PRE**	**Births**	**CHD**	**PRE**	**Births**	**CHD**	**PRE**	
2005	19,467	43	2.21	27,835	29	1.04	47,302	72	1.52	52.83
2006	18,957	58	3.06	3,1007	53	1.71	49,964	111	2.22	44.13
2007	25,889	84	3.24	35,782	69	1.93	61,671	153	2.48	40.57
2008	24,475	85	3.47	35,329	73	2.07	59,804	158	2.64	40.50
2009	26,593	90	3.38	40,145	76	1.89	66,738	166	2.49	44.06
2010	36,732	142	3.87	44,228	100	2.26	80,960	242	2.99	41.51
2011	36,700	149	4.06	44,722	115	2.57	81,422	264	3.24	36.66
2012	39,763	195	4.90	49,291	158	3.21	89,054	353	3.96	34.64
2013	35,551	178	5.01	41,907	132	3.15	77,458	310	4.00	37.09
2014	50,363	208	4.13	58,848	152	2.58	10,9211	360	3.30	37.46
2015	35,959	154	4.28	40,481	113	2.79	76,440	267	3.49	34.82
2016	62,281	420	6.74	69,238	326	4.71	131,519	746	5.67	30.18
2017	49,217	300	6.10	53,464	230	4.30	102,681	530	5.16	29.42
2018	49,853	300	6.02	52,224	250	4.79	102,077	550	5.39	20.45
2019	49,134	259	5.27	53,128	221	4.16	102,262	480	4.69	21.09
2020	40,445	230	5.69	43,366	188	4.34	83,811	418	4.99	23.77
Total	60,1379	2,895	4.81	72,0995	2,285	3.17	132,2374	5,180	3.92	34.17

* *Cochran-Armitage trend (CAT) P < 0.05*.

$ *Cochran-Armitage trend (CAT) P < 0.05*.

& *Cochran-Armitage trend (CAT) P < 0.05*.

# *Cochran-Armitage trend (CAT) P < 0.05*.

*Extent%Δ = (the CHD prevalence in urban areas- the CHD prevalence in rural areas)/ the CHD prevalence in urban areas ×100%*.

### The Prevalence of CHD Between Urban Areas and Rural Areas in Jinan From 2005 to 2020

From 2005 to 2020, the CHD prevalence was 4.81 per 1,000 births in urban areas and 3.17 per 1,000 births in rural areas. Time trend analysis showed an upward trend of the CHD prevalence in urban and rural areas, and respectively increased by 157.45% and 316.03% in 2020 compared with 2005. Obviously, the increasing range in rural areas was larger than that in urban areas.

The gap of CHD prevalence between urban and rural areas was analyzed. Overall, the CHD prevalence in urban areas was 34.17% higher than that in rural areas, but the gap, gradually, was narrowed, from 52.83% in 2005 to 23.77% in 2020 (Table 1, Figure 1).

### The Proportion of CHD Before 28 Gestation Weeks and After 28 Gestation Weeks in Jinan From 2005 to 2020

Between 2005 and 2020, the proportion of CHD was 31.20% before 28 gestation weeks and 68.80% after 28 gestation weeks. Time trend analysis showed an upward trend of the CHD proportion before 28 gestation weeks and increased by 192.82% in 2020 compared with 2005. On the contrary, a downward trend of the CHD proportion was found after 28 gestation weeks, which decreased by 31.11% in 2020 compared with 2005.

**Figure 2:**
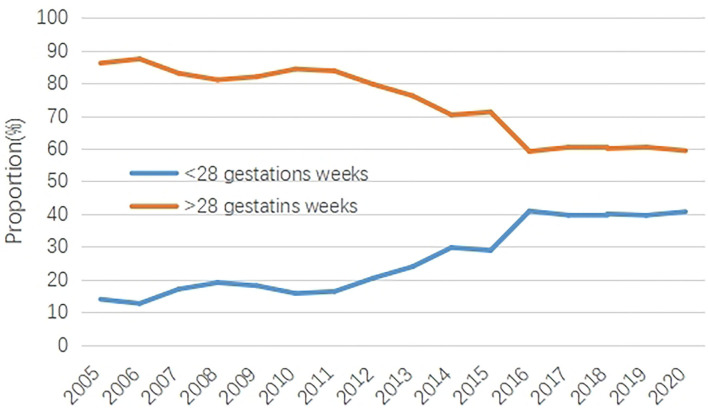
The proportion of CHD before 28 gestation weeks and after 28 gestation weeks from 2005 to 2020.

Overall, the gap of the CHD proportion between before 28 gestation weeks and after 28 gestation weeks gradually narrowed, from 520% in 2005 to 45.88% in 2020 (Table 2, Figure 2).

**Table 2:** The propotion of CHD before 28 gestation weeks and after 28 gestation weeks from 2005 to 2020.

**Year**	**Births**	Before 28 gestation weeks^*^		**After 28 gestation weeks^#^**		**Extent%Δ^$^**
		**CHD**	**Propotion (%)**	**CHD**	**Propotion (%)**	
2005	47,302	10	13.89	62	86.11	520.00
2006	49,964	14	12.61	97	87.39	592.86
2007	61,671	26	16.99	127	83.01	388.46
2008	59,804	30	18.99	128	81.01	326.67
2009	66,738	30	18.07	136	81.93	353.33
2010	80,960	38	15.70	204	84.30	436.84
2011	81,422	43	16.29	221	83.71	413.95
2012	89,054	72	20.40	281	79.60	290.28
2013	77,458	74	23.87	236	76.13	218.92
2014	109,211	107	29.72	253	70.28	136.45
2015	76,440	77	28.84	190	71.16	146.75
2016	131,519	305	40.88	441	59.12	44.59
2017	102,681	210	39.62	320	60.38	52.38
2018	102,077	220	40.00	330	60.00	50.00
2019	102,262	190	39.58	290	60.42	52.63
2020	83,811	170	40.67	248	59.33	45.88
Total	132,2374	1,616	31.20	3,564	68.80	120.54

* *Cochran-Armitage trend (CAT) P < 0.05*.

# *Cochran-Armitage trend (CAT) P < 0.05*.

$ *Cochran-Armitage trend (CAT) P < 0.05*.

*Extent%Δ=(the CHD propotion after 28 gestation weeks- the CHD propotion before 28 gestation weeks)/ the CHD propotion after 28 gestation weeks ×100%*.

### The Difference of CHD Prevalence in Jinan According to Different Maternal Characteristics

**Figure 3:**
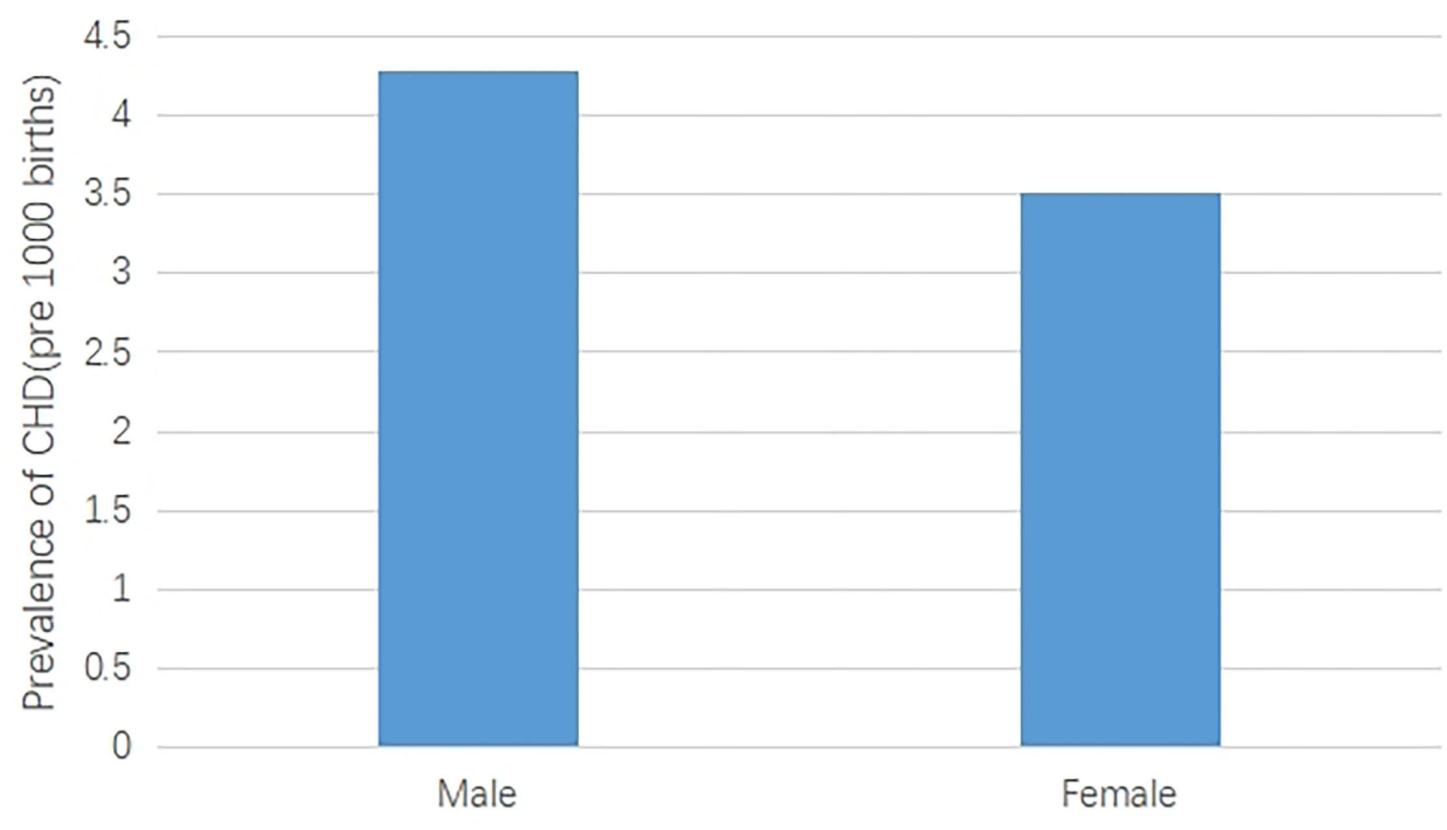
The prevalence of CHD between male births and female births.

Firstly, the CHD prevalence of male births was 17.92% higher than that of female births, and this difference was statistically significant (*p* < 0.05). Secondly, the CHD prevalence of maternal delivery season was highest in autumn (4.73 per 1,000 births) and least in spring (3.49 per 1,000 births), and the difference was statistically significant (*p* < 0.05). Besides, significant differences were also found in different maternal age groups. The highest CHD prevalence was found in over 35 years old (7.18 per 1,000 births), followed by under 20 years old (5.52 per 1,000 births) (Table 3, Figure 3).

**Table 3:** The prevalence of CHD from 2005 to 2020 in Jinan according to different characteristics.

**Variable**	**Births**	**CHD**	**Prevalence**	***P* value**
**Infant gender**				<0.001
Male	695,665	2,978	4.28	
Female	626,709	2,202	3.51	
**Season**				<0.001
Spring (March, April, May)	320,421	1,117	3.49	
Summer (June,July,August)	320,452	1,134	3.54	
Autumn (September, October.	326,300	1,544	4.73	
November)				
Winter (December,January,February)	355,201	1,385	3.90	
**Maternal age**				<0.001
<20	17,204	95	5.52	
20–24	235,339	736	3.13	
25–29	519,356	1,730	3.33	
30–34	388,498	1,456	3.75	
35-	161,977	1,163	7.18	

### The Prevalence of the 5 Most Common CHD Subtypes in Jinan From 2005 to 2020

Overall, atrial septal defect (ASD), patent ductus arteriosus (PDA), ventricular septal defect (VSD), tetralogy of Fallot (TOF), and atrioventricular septal (AVSD) were the 5 most common subtypes. The prevalence of these CHD subtypes was 3.07 per 1,000 births, 1.62 per 1,000 births, 1.18 per 1,000 births, 0.62 per 1,000 births, and 0.47 per 1,000 births, respectively.

Time trend analysis showed that the total CHD prevalence in different subtypes increased over time. Compared with 2005, the 5 most common CHD subtypes, respectively, increased by 251.51, 162.74, 109.23, 33.08, and 15.32%. Obviously, the increasing range of ASD was largest among the 5 most common CHD subtypes. Besides, we also found that ASD, PDA, VSD, and TOF had the highest prevalence in 2016 from 2005 to 2020 (Table 4).

**Table 4:** The prevalence of the the 5 most common CHD subtypes in Jinan from 2005 to 2020.

**Year**	Atrial septal defect^*^		**Patent ductus arteriosus^#^**		**Ventricular septal defect^&^**		**Tetralogy of Fallot^$^**		**Atrioventricular septal^@^**	
	**CHD**	**PRE**	**CHD**	**PRE**	**CHD**	**PRE**	**CHD**	**PRE**	**CHD**	**PRE**
2005	17	0.87	12	0.62	11	0.57	9	0.46	8	0.41
2006	28	1.48	11	0.58	20	1.06	9	0.47	10	0.53
2007	35	1.35	17	0.66	27	1.04	12	0.46	11	0.42
2008	42	1.72	19	0.78	35	1.43	14	0.57	8	0.33
2009	41	1.54	21	0.79	34	1.28	13	0.49	10	0.38
2010	79	2.15	43	1.17	33	0.90	21	0.57	19	0.52
2011	100	2.72	57	1.55	40	1.09	20	0.54	20	0.54
2012	114	2.87	75	1.89	41	1.03	26	0.65	21	0.53
2013	107	3.01	57	1.60	42	1.18	21	0.59	19	0.53
2014	135	2.68	77	1.53	43	0.85	25	0.50	16	0.32
2015	98	2.73	65	1.81	25	0.70	20	0.56	13	0.36
2016	292	4.69	167	2.68	99	1.59	51	0.82	34	0.55
2017	205	4.17	95	1.93	71	1.44	31	0.63	25	0.51
2018	211	4.23	98	1.97	62	1.24	33	0.66	21	0.42
2019	192	3.91	85	1.73	65	1.32	35	0.71	22	0.45
2020	150	3.71	75	1.85	63	1.56	30	0.74	28	0.69
Total	1846	3.07	974	1.62	711	1.18	370	0.62	285	0.47

* *Cochran-Armitage trend (CAT) P < 0.05*.

# *Cochran-Armitage trend (CAT) P < 0.05*.

& *Cochran-Armitage trend (CAT) P < 0.05*.

$ *Cochran-Armitage trend (CAT) P < 0.05*.

@ *Cochran-Armitage trend (CAT) P < 0.05*.

## Discussion

The total CHD prevalence in Jinan was 3.92 per 1,000 births from 2005 to 2020 and 5.61 per 1,000 births from 2016 to 2020, which were roughly equivalent to the CHD prevalence in China (13) over the same period but were higher than that in Changzhou (14), and lower than that in Zhejiang (15), in Asia, and in the world (2). We speculated that there may be several reasons for this difference. Firstly, it might be due to the different monitored periods. The monitored period, in our study, was from 12 gestation weeks to 7 days after births, but that in Asia or in the world was from birth to 6 years old, and that in Changzhou was from 28 gestation weeks to 7 days after births. Secondly, various screening approaches also played an important role. In our study, B-ultrasound was the main screening method for CHD in Jinan. However, in the study in Zhejiang, in addition to B-ultrasound examination, the pulse oximetry was used for CHD screening after birth.

In our study, 5 epidemiological characteristics of congenital heart disease in Jinan were found.

Firstly, the total CHD prevalence increased significantly over time in Jinan; the upward trend was also found in other studies in China (16, 17). We found an “S” shape in the time trend of the CHD prevalence, and 2011 and 2016 were two obvious growth inflection points. In particular, the prevalence of atrial septal defect (ASD) and ventricular septal defect (VSD) reached their highest levels in 2016 in nearly 15 years. The phenomenon was explained by the following two reasons. On the one hand, it might be attributed to the established information systems. Jinan Maternal and Child Health Information System was an information system based on case collection. Since 2011, every examination of pregnant women throughout pregnancy, especially ultrasound examination, can be found in the information system. However, previous information collections were aggregated data, and information omissions or misdiagnosis of CHD existed. On the other hand, the comprehensive two-child policy implemented in 2016 had a significant impact on the increase in the prevalence of CHD (18, 19). After the implementation of the policy, women over 35 years old in Jinan increased by 45% (20), which undoubtedly increased the prevalence of CHD.

Secondly, we found that the proportion of CHD before 28 gestation weeks increased gradually. This phenomenon was also found in other studies on the CHD diagnosis gestational weeks (21). Large-scale B-ultrasound examination was an important reason for this phenomenon. After 2016, free prenatal ultrasound was included in public health projects in Jinan. Every pregnant woman can obtain a free prenatal ultrasound examination at about 24 gestation weeks, and more and more CHD can be found in the perinatal period, resulting in increasing prevalence.

**Figure 4:**
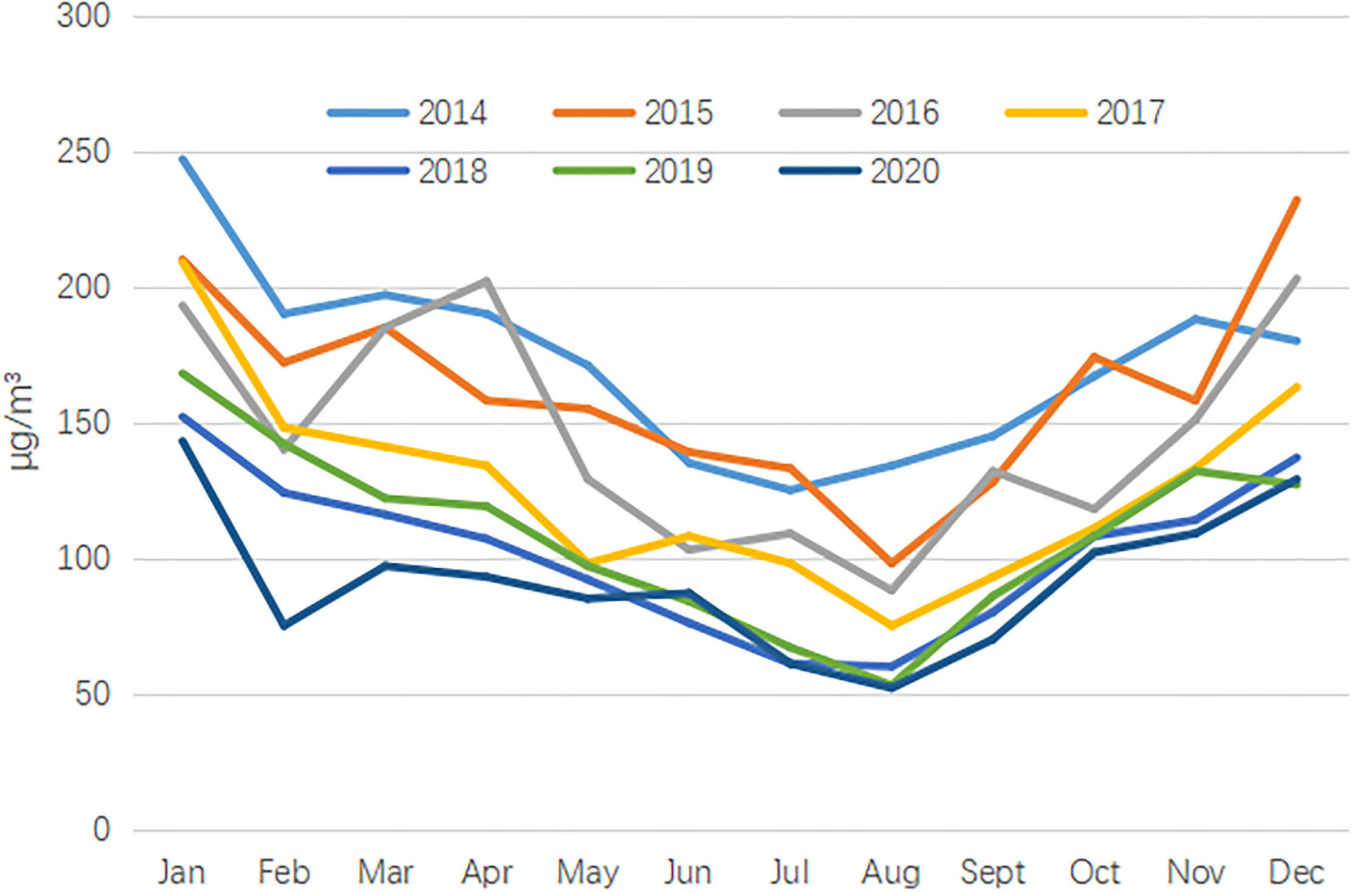
The number of PM10 in different months from 2014 to 2020.

**Figure 5:**
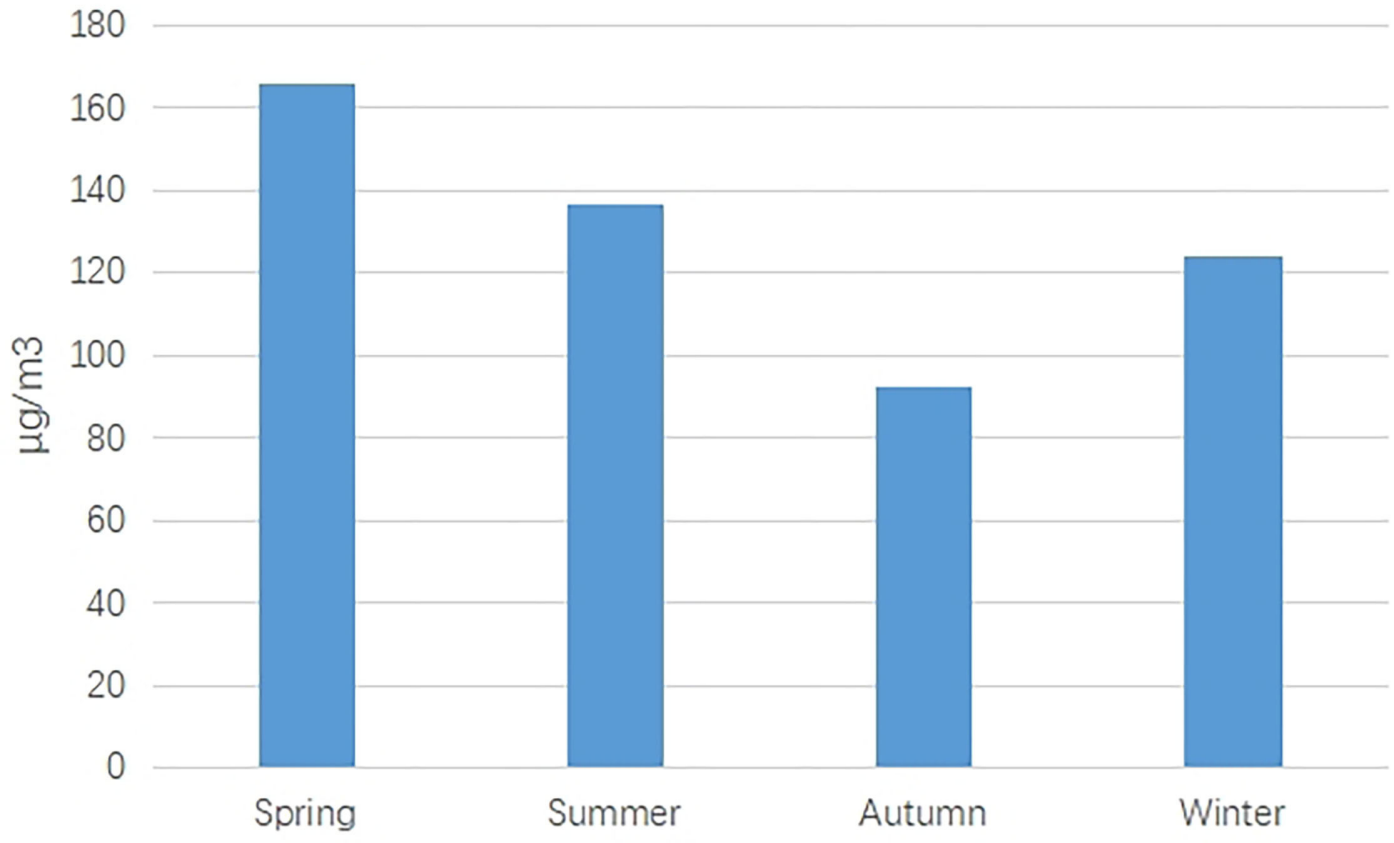
The number of PM10 in different seasons.

Thirdly, significant differences across gender, maternal delivery season, and maternal age were found. (i) The CHD prevalence in male births was higher than that in female births, which was consistent with some previous studies (22, 23). This difference may be explained by the higher susceptibility of Y chromosome than X chromosome (24). (ii) The CHD prevalence in autumn was highest. As we all know, the critical period of fetal heart and embryo development was 3–8 gestation weeks. In our study, the CHD prevalence in autumn was highest, which indicated that early maternal exposure was in winter. In Jinan, air pollution was relatively serious in winter, especially PM10 (Figures 4, 5). Some studies have pointed out that PM2.5 or PM10 was an important risk factor in birth defects (25, 26). (iii) Like other studies, we also found high CHD prevalence in elderly pregnant women (27–29). As more and more women delay their childbearing age, the government should attach great importance to the above issue. It is estimated that the maternal age has delayed from 26.12 years old in 2005 to 31.26 years old in 2020.

Fourthly, the total CHD prevalence in urban areas was higher than that in rural areas, which was also reported in many other pieces of literature (30, 31). Perhaps, the stronger overall health awareness, the wider use of prenatal diagnosis techniques, more accessibility, and reporting practices in urban areas can be used to explain the phenomenon (32). Interestingly, the gap of the CHD prevalence between urban areas and rural areas was getting smaller and smaller. Several reasons can be used to explain this phenomenon. (i) After 2011, with the advancement of public health projects, the use of prenatal diagnostic techniques became more widespread in rural areas, allowing a large number of congenital heart diseases to be detected prenatally. (ii) An important reason for the phenomenon was changes in environmental exposures. In recent years, the environmental protection measures implemented in urban areas of Jinan have become more and more strict, and the environmental quality has become better and better. On the contrary, the environmental quality in rural areas has not been greatly improved. Especially during the Spring Festival, due to the use of firecrackers, the environmental quality in rural areas was very poor (33). National Environmental Monitoring Center reports that more than 60% cities in rural areas have excessive air pollution during the Spring Festival holiday (34), and the data in our study also showed the poor air quality in January, each year during the Spring Festival from 2014 to 2018.

Fifthly, in Jinan, the reported CHD was dominated by mild lesions (84.31%), such as VSD, ASD, and PDA. The finding also was confirmed in other studies (35). The following reasons can explain this phenomenon. Firstly, comprehensive use of echocardiography and improved echocardiographic techniques were likely to account substantially for the increased prevalence of mild lesions. With the improvement of health awareness, more and more pregnant women were checked fetal health by B-ultrasound. In 2020, the proportion of fetal cardiac ultrasound examination reached 92.13%. So, many previous mild lesions were reported in the prenatal period. The second reason should be attributed to air pollution. The terrain in Jinan belonged to a basin bottom structure, which made the air pollutants, especially PM10, unable to be dissipated in time. Some epidemiologic studies have suggested the possible association of maternal exposure to PM10 during the first trimester of gestation with VSD, ASD, and PDA (25, 36).

### Limitation

Firstly, the prevalence of CHD in our study was based on hospitals; births with CHD given out of hospital would be missed. Therefore, the actual prevalence of CHD might be underestimated. Secondly, as a cross-sectional study, some risk factors of CHD, such as the natural environment or other maternal complications, were not considered because of limited data.

## Conclusion

The CHD prevalence was increased in Jinan from 2005 to 2020. The CHD prevalence in urban areas and before 28 gestation weeks was increased faster. Mild lesions, such as VSD, ASD, and PDA, were the dominating subtypes. Elderly pregnant women and women in rural areas should be focused.

## Data Availability Statement

The original contributions presented in the study are included in the article/supplementary material, further inquiries can be directed to the corresponding author.

## Ethics Statement

The studies involving human participants were reviewed and approved by Ethics Committee of Jinan Maternity and Child Care Hospital Affiliated to Shandong First Medical University. The patients/participants provided their written informed consent to participate in this study.

## Author Contributions

LZ, BL, and HL participated in data collection. ZL conceived the idea, participated in data analysis, and drafted the manuscript. CW and SY participated in the design and implementation of the study. All authors read and approved the final manuscript.

## Funding

This investigation was supported by the Shandong province medical and health science development project (2018WS488) and the Jinan medical and health science development project (2018-1-29).

## Conflict of Interest

The authors declare that the research was conducted in the absence of any commercial or financial relationships that could be construed as a potential conflict of interest.

## Publisher's Note

All claims expressed in this article are solely those of the authors and do not necessarily represent those of their affiliated organizations, or those of the publisher, the editors and the reviewers. Any product that may be evaluated in this article, or claim that may be made by its manufacturer, is not guaranteed or endorsed by the publisher.
